# Rethinking Japan’s Infallibility Principle for a Better Pandemic Response

**DOI:** 10.7759/cureus.39270

**Published:** 2023-05-20

**Authors:** Yudai Kaneda, Akihiko Ozaki, Tetsuya Tanimoto

**Affiliations:** 1 School of Medicine, Hokkaido University, Sapporo, JPN; 2 Breast and Thyroid Surgery, Jyoban Hospital of Tokiwa Foundation, Iwaki, JPN; 3 Internal Medicine, Navitas Clinic, Kawasaki, JPN

**Keywords:** transparency in healthcare policy, bureaucratic culture, principle of infallibility, covid-19, japan

## Abstract

The "infallibility principle" in Japanese government bureaucracy has led to conservative responses in handling the COVID-19 pandemic, with adherence to initial methods, like the 3Cs (crowded places, close-contact settings, and confined and enclosed spaces), and resistance to policy changes, despite evolving scientific findings on airborne transmission. This inflexible approach resulted in multiple states of emergency, social and economic losses, and increased health challenges. Although claims have been made of near-total control by May 2022, lack of sufficient verification and the record death toll in the eighth wave in the fall of 2022 suggest a reactionary rather than proactive policy approach.

## Editorial

The Japanese government bureaucracy has been criticized for its implicit adherence to the "infallibility principle," a principle that the policy enforcement organization must not entertain the concept of failure and has no tolerance for it [1]. Consequently, Japanese civil servants often cling to the precedent with conservative attitudes, recognizing that risk-taking and alterations may yield negative personnel assessment and hamper their career advancement in case of failure [1].

In the assessment of Japan’s COVID-19 pandemic response, justification based on the principle of infallibility has been particularly evident for implemented policies. Since metro rails, air-conditioned buses, and air-conditioned malls are increasing worldwide, scientific discussion on airborne transmission has been emphasized overseas, and ventilation has been the primary measure against aerosol infection of SARS-CoV-2 [2]. In contrast, Japan's approach centered on minimizing transmission risk through the 3Cs (crowded places, close-contact settings, and confined and enclosed spaces) and prioritized reducing human contact based on the initial observations at the beginning of the COVID-19 pandemic [3]. This approach led to six declarations of a state of emergency and semi-emergency coronavirus measures between 2020 and 2022, urging residents in impacted areas to stay home, businesses to limit hours or close, and public events to face restrictions. These actions resulted in the loss of various social experiences, physical and mental health challenges due to immobilization and isolation, and economic losses in Japanese society [4].

Nevertheless, Japanese medical experts have claimed that Japan informed its citizens about SARS-CoV-2 aerosol transmission more swiftly than other G7 (Group of Seven) countries, reducing damage and achieving near-total control by May 2022 [5]. In reality, however, such a claim is not based on ample verification nor theoretical backgrounds, as illustrated by a futile national database called "HER-SYS," which was introduced by the Japanese government to monitor infection trends and was not publicly accessible due to security reasons. This was in contrast to the self-reflexive approach of governments such as the United Kingdom, which acknowledged the failure of their previous policies in light of accumulated scientific findings. Consequently, vital decisions to revise policies in Japan have been postponed, yielding reactive rather than proactive responses. Indeed, without infection control revisions, the situation deteriorated, leading to the highest recorded death toll during the eighth wave since the fall of 2022, surpassing 12,000 only in one and a half months.

Of note, the infallibility principle is followed by most countries, including India, for political benefit. However, in order to improve the response to possible future pandemics other than COVID-19, such as avian influenza, and to protect the health of the public, each government should shift from the infallibility principle to a transparent, data-driven approach. This shift entails encouraging open dialogue among diverse stakeholders, prioritizing objective data for policy decisions, ensuring transparent communication, and embracing third-party evaluations to admit and allow failures for improvement. By recognizing the need for change and learning from other countries' experiences, countries that adhere to the infallibility principle, including Japan, can develop more effective, evidence-based policies characterized by improved public health, increased societal resilience, and strengthened international collaboration. By recognizing the need for change and learning from other countries' experiences, countries that adhere to the infallibility principle, including Japan, can develop more effective, evidence-based policies characterized by improved public health, increased societal resilience, and strengthened international collaboration.
